# Study of SH-SY5Y Cancer Cell Response to Treatment with Polyphenol Extracts Using FT-IR Spectroscopy

**DOI:** 10.3390/bios7040057

**Published:** 2017-11-30

**Authors:** Valerio Ricciardi, Marianna Portaccio, Simona Piccolella, Lorenzo Manti, Severina Pacifico, Maria Lepore

**Affiliations:** 1 Dipartimento di Fisica “Ettore Pancini”, Università di Napoli “Federico II”, 80126 Napoli, Italy; val.ricciardi89@gmail.com (V.R.); lorenzo.manti@na.infn.it (L.M.); 2 Dipartimento di Medicina Sperimentale, Università della Campania “Luigi Vanvitelli”, 80138 Napoli, Italy; marianna.portaccio@unicampania.it; 3 Dipartimento di Scienze e Tecnologie Ambientali Biologiche e Farmaceutiche, Università della Campania “Luigi Vanvitelli”, 81100 Caserta, Italy; simona.piccolella@unicampania.it (Si.P.); severina.pacifico@unicampania.it (Se.P.)

**Keywords:** plant polyphenols, neuroblastoma cells, Fourier-Transform Infrared microspectroscopy, cells-polyphenols interaction

## Abstract

Plant polyphenols are important components of human diet and a number of them are considered to possess chemo-preventive and therapeutic properties against cancer. They are recognized as naturally occurring antioxidants, but also as pro-oxidant, pro-apoptotic, or chromosomal aberrations inducers, depending on their concentration and/or the stage of cell-cycle of the cells with which they interact. For these reasons, particular interest is devoted to knowing the total effects of polyphenols on the cell cycle and metabolism. Fourier-Transform Infrared (FT-IR) spectroscopy thanks to its ability in analyzing cells at a molecular level can be particularly useful in investigating the biochemical changes induced in protein, nucleic acid, lipid, and carbohydrate content of cells by means of polyphenols administration. Spectroscopic analysis was performed on *in vitro* human SH-SY5Y neuroblastoma cells that were exposed to different doses of a cherry derived polyphenol extract. The infrared spectra that were obtained from unexposed and exposed cells show significant differences that can be helpful in order to understand the cells-polyphenols interaction.

## 1. Introduction

Overproduction of oxidants that overwhelm the cellular antioxidant capacity results in oxidative stress, a deleterious and pathogenic process that can be an important mediator of damage to cell structures, including lipids, membranes, proteins, and DNA. On the other hand, cells and organisms are constantly exposed to some oxidizing agents, some of which are necessary for life. To sustain optimal physiologic conditions in the body, the key factor is to maintain a balance between oxidants and antioxidants [1,2].

Epidemiological evidence has highlighted a strong correlation between plant metabolites having bioactive properties and human health. In particular, edible plants, which counteract free radical overproduction during oxidative stress, are considered as an effective tool to prevent several diseases, and indeed to increase the well-being. Plant polyphenols are important components of human diet and a number of them are considered to possess chemo-preventive and therapeutic properties against cancer. They are recognized as naturally occurring antioxidants but also as pro-oxidant, pro-apoptotic, or chromosomal aberrations inducers, depending on their concentration and/or the stage of cell-cycle of the cells with which they interact. Like antioxidants, polyphenols may improve cell survival, conversely, as pro-oxidants, they may induce apoptosis and prevent tumour growth [3,4].

**Figure 1:**
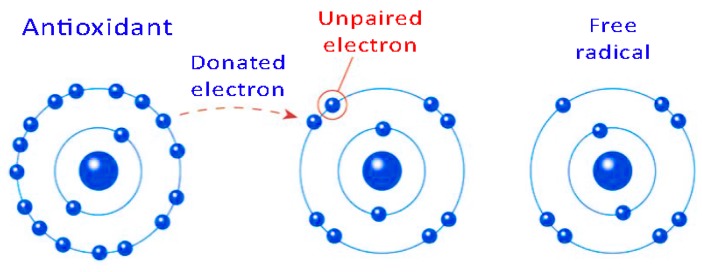
Schematic representation of the inactivation mechanism of reactive free radicals.

Polyphenols are organic molecules, which are widely present in the plant kingdom that can be classified into different groups based on the number of phenol rings contained and the structural elements that bind these rings to one another. The term ‘phenol’, which is chemically referred to a chemical compound obtained by replacing one hydrogen in an aromatic hydrocarbon with a hydroxy (-OH) group, is currently extended to any substance that possesses an aromatic ring bearing one or more hydroxy substituents, including functional derivatives. Phenolic compounds are efficacious antioxidants, which, through hydrogen atom or electron transfer mechanisms, could neutralize dangerous free radicals, exhibiting radical scavenging capacity (RSC) (Figure 1).

**Figure 2:**
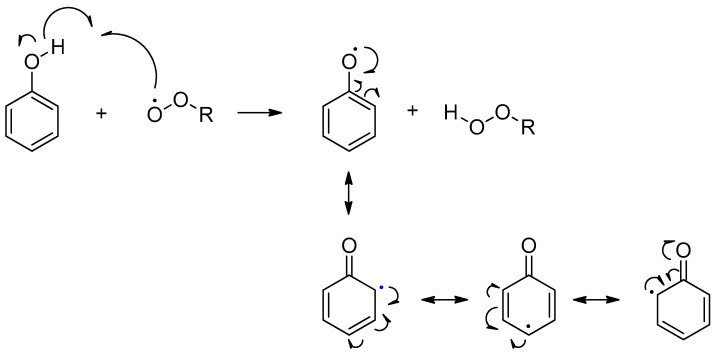
Schematic representation of resonance-stabilized phenoxyl radicals by hydrogen atom transfer from hydroxyl group in phenol to free radicals.

The hydrogen atom transfer from the phenol ring to free radicals, such as peroxyl radicals (ROO^•^), results in a phenoxyl radical stabilized by resonance delocalization (Figure 2).

The extract of polyphenols that is used in this work is derived from autochthonous sweet cherries of the Campania Region (Italy). In preliminary measures [5], it showed to act as reactive oxygen species scavengers at low concentrations, while showing pro-oxidant activity at higher concentrations [6,7].

In the present work, we report the results of an investigation on the effects of this polyphenol extract on neuroblastoma cells by using Fourier-Transform Infrared (FT-IR) spectroscopy. FT-IR spectroscopy is a powerful analytical method for the detection of molecular and structural changes in a large class of chemical and biological samples [5,8,9,10,11]. Some authors proposed to use infrared spectroscopy for investigating the effects of polyphenolic compounds on cells and tissues [12,13,14,15,16,17]. Lipid peroxidation and protein phosphorylation as consequences of the intensified production of reactive oxygen species were investigated in monkey fibroblast cells by Vileno [12], and on lipids that were extracted from human plasma by Olenko et al. [13]. Cakmak et al. [14] used FT-IR spectra to observe differences that were induced in rainbow trout liver cells by different polyphenol concentrations. Derenne et al. [15] studied the metabolic changes that were induced by six types of polyphenols on T98G glioma cells using FT-IR spectroscopy combined with principal component analysis (PCA). Barraza-Garza et al. [16] used infrared spectra to evaluate the antioxidant activity of polyphenols on rat enterocytes using a ratiometric approach. Ulrich [17] proposed FT-IR as an analytical technique to study the polyphenol-protein interaction.

In this study, we analyzed the infrared spectra of SH-SY5Y neuroblastoma cells that were exposed to two different doses (25 μg/mL and 500 μg/mL) of the cherry polyphenol extract using two different exposure times (48 and 72 h) by assigning and evaluating the absorption bands. We also analyzed the differences in infrared spectra between control cells (not exposed to polyphenol extract) and exposed cells by considering peak positions and intensities. In addition, we calculated the ratios of the areas of the bands corresponding to proteins, lipids, and nucleic acids [18]. We also performed a detailed analysis of the secondary structure of protein content in different cases by using Amide I band deconvolution [19]. The obtained results showed that FT-IR could be a useful tool for studying the complex cell-polyphenols interaction process.

## 2. Materials and Methods

### 2.1. Cell Line

**Figure 3:**
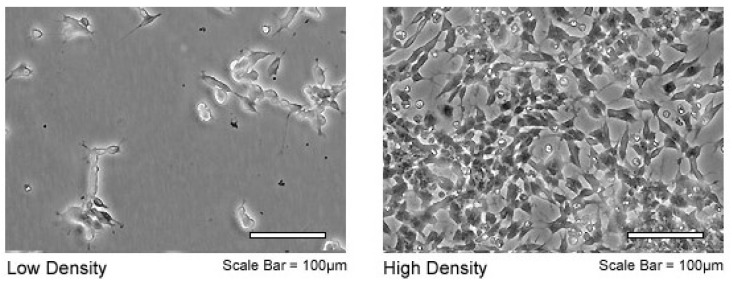
SH-SY5Y cells at different culture density (TCC, American type culture collection).

The cell line that was used for all the analyses of this study is the SH-SY5Y neuroblastoma (American Type Culture Collection, Manassas, VA, USA) (Figure 3). This cell line was chosen because it is representative of the most common cancer in infants and the third most common cancer in children, after leukaemia and brain cancer. Moreover, it is particularly important in neuroprotection studies to develop new strategies for the treatment and/or prevention of central nervous system disorders [20,21,22]. SH-SY5Y is a cell line that is subcloned from a bone marrow biopsy taken from a four-year-old female with neuroblastoma. These cells are often used as in vitro models of neuronal function and differentiation. Cells were cultured in vitro in DMEM medium with the addition of 20% fetal bovine serum, 1% of penicillin and 1% of l-glutamine.

### 2.2. Preparation of Cherry Derived Polyphenol Extract

The polyphenol extract that was used in this study was prepared from sweet cherry (*Prunus avium* L. cv. Della Recca) fruits, which was collected from trees grown under standard commercial practices and trained in the same experimental orchard located at Pignataro Maggiore (Caserta, Southern Italy) owned by the C.R.A. Research Unit on Fruit Trees. After harvest, the fruits were immediately transported to the laboratory, where they were screened for uniformity, appearance, and the absence of physical defects or decay. Three replicate samples (100.0 g each) of the cherries were selected, cleaned with MilliQ water, drained, pitted, and then ground in a porcelain mortar and pestle chilled with liquid N_2_, until particles of homogeneous size were obtained; frozen powdered cherry samples were lyophilized using an FTS-System Flex-DryTM instrument (SP Scientific, Stone Ridge, NY, USA). Three samples of cherries (≈5.0 g each) underwent ultrasound assisted maceration (UAM; Advantage Plus model ES, Darmstadt, Germany) using pure water as extracting solvent (three extraction cycles; 30 min × 150.0 mL each); after centrifugation, supernatants were pooled and dried under vacuum by a rotary evaporator (Heidolph Hei-VAP Advantage (Schwabach, Germany), to yield to crude extract (PaDRw). In order to unravel the metabolic composition of the aqueous extract, three aliquots of them (≈0.7 g each) were afterwards chromatographed on Amberlite XAD-4, eluting with water first (PaDRw-1), and then with MeOH (PaDRw-2). Quantitative analysis demonstrated that about 63% of the whole PaDRw extract was constituted by hexitol, followed by ≈22.8% of fructose and ≈10.7% of glucose; phenol compounds, mainly chlorogenic acids and flavonoids, accounted only for about 2.2%.

In the present work, two different concentrations of the crude extract (25 µg/mL and 500 µg/mL) were used. These two doses were chosen because it is suggested that at concentration >200 μg/mL the cytoprotective scavenging activity may turn into pro-oxidant cytotoxicity [23,24].

### 2.3. Preparation of Cherry Derived Polyphenol Extract

**Figure 4:**
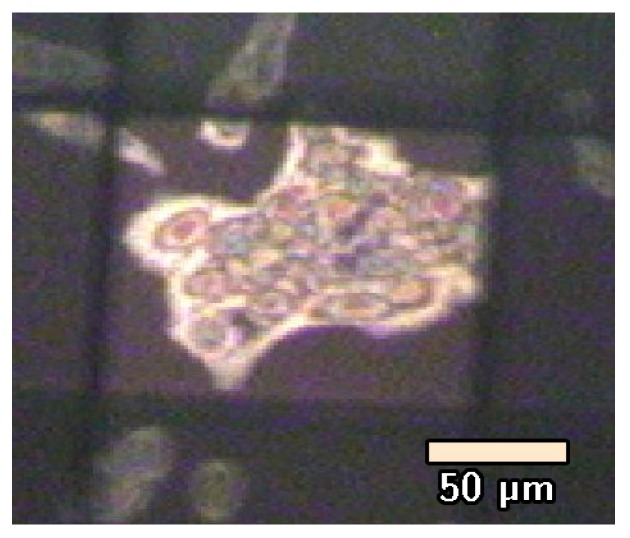
Micrograph at 10× magnification of SH-SY5Y cells control sample adherent to the MirrIR slide. A cells cluster is visible in the brighter area that is manually selected for collecting the signal for Fourier-Transform Infrared (FT-IR) spectroscopy.

The cells were seeded on MirrIR slide (25 × 25 mm^2^) (Kevley Technologies, Chesterland, OH, USA), a specific reflection FT-IR spectroscopy microscope slide, nested in Petri dish capsules (d = 35 mm). After the time that was required for the cells to adhere properly to the slide, the Petri were removed from incubation, and, for some of them, PaDRw was dissolved in the cell culture medium, at two different concentrations 25 µg/mL (PaDRw-25) and 500 µg/mL (PaDRw-500). Petri capsules were then immediately re-stored in an incubator and the extracts were left to act for 48 or 72 h. Every sample was prepared in triplicate. The slides were seeded with a cell surface density that was equal to

(1) $$\sigma =\frac{C}{S}=\frac{C}{\pi r^{2}}=\frac{10^{5}}{\pi \left(1.75\;{\mathrm{cm}}^{2}\right)}\;\approx \;\frac{10,000\;\mathrm{cells}}{{\mathrm{cm}}^{2}}$$

where *S* is the surface area of the Petri dish for a total of approximately 5 × 10^6^ cells/Petri (Figure 4). This cell density was chosen because it guarantees both the presence of inter-cell space for the measurement of the background signal without affecting cell survival and both the presence of clusters of cells that are necessary to obtain a sufficiently intense signal.

### 2.4. Cell Fixing Protocol

Within the time specified previously, cells were removed from the growth medium and fixed in a 3.7% formaldehyde PBS solution for 20 min at room temperature, and, then, briefly washed in distilled water for 3 s to remove the residue PBS from the surface of the cells. Subsequently, µFT-IR samples were dried under ambient conditions and stored in a desiccator until analysis (water molecules have a strong IR signal so the humidity of the samples must be kept under control). The possibility of analyzing FT-IR spectra of fixed cells rather than in vivo is considered in different works reported in the literature. The choice to work with these samples is much more convenient because the fixing procedure preserves the biochemical properties of the cells during the transport and the measurement phase [25,26,27].

### 2.5. Spectra Acquisition

The instrument that was used for the acquisition of the IR absorption spectra of the cell samples was a Spectrum One FTIR (PerkinElmer, Shelton, CT, USA) spectrometer, equipped with a Perkin Elmer Multiscope system infrared microscope and an MCT (mercury cadmium telluride) detector. The measurements were performed on cells adherent on MirrIR slides having an area of 25 × 25 mm^2^ in transflection mode. The background signal was acquired in a region of the MirrIR slide that was free of cells. For each experimental condition, three slides were prepared. Every slide was examined in different regions of about 100 × 100 μm size (see Figure 4), and three spectra were acquired for each position. The measurements were made at room temperature by collecting the signal in the spectral region between 4000 and 800 cm^−1^ using 64 scans with a spectral resolution of 4 cm^−1^ and a 5 s acquisition time for each spectrum.

### 2.6. Data Analysis

The spectra were preliminarily analyzed using the application routines provided by the software package (“Spectrum” Perkin Elmer Inc., Hopkinton, MA, USA) controlling the whole data acquisition system. 

In order to highlight the similarities between spectra obtained on cells in the same experimental conditions, and to emphasize the differences between spectra for cells that were subjected to different doses of polyphenols, we adopted a univariate analysis that was already used in Refs. [28,29].

In particular, for each wavenumber point, the intensity of the spectrum (*y_i_* variable) was compared to the corresponding value of another spectrum variable (*x_i_* variable) referring to a different sample, by performing a linear regression or univariate analysis of data. The basic assumption is that the spectrum Y (containing the n data *y_i_*) is a linear function of another spectrum X (containing the n data *x_i_*), that is $y_{i}=\left(mx_{i}+p+\varepsilon_{i}\right)$. If no structural change occurs in the sample, then the perturbation terms *ε_i_* depends only on the experimental conditions and follows a Gaussian distribution, with mean equal to zero. Regression analysis allows us to determine the parameters *m* and *p*. To evaluate the similarity between data sets, we calculated the sample coefficient of determination *R*^2^, defined as

$$R^{2}=1-\frac{\sum^{\text{​}}{\left[y_{i}-\left(mx_{i}+p\right)\right]}^{2}}{\sum^{\text{​}}{\left[y_{i}-\bar{y}\right]}^{2}}$$

where $\bar{y}$ is the average value of vector Y. $R^{2}$ ranges from 0 for uncorrelated data to 1 for perfect linear dependence. The linear regression was evaluated for a number of points ranging between 500 and 700, implying a high level of significance of the procedure. This analysis was performed with MATLAB (Version 8.3, MathWorks, Natick, MA, USA) program.

The spectra were further analyzed in terms of convoluted peak functions to determine the basic vibrational modes that contribute to the FT-IR signal by using a best-fit peak fitting routine of Origin software (Version 9.0, OriginLab Corporation, Northampton, MA, USA), based on the Levenberg-Marquardt nonlinear least-square method. Lorentzian curves were used. Peaks constituting the spectrum were manually selected in order to define the starting conditions for the best-fit procedure. The best-fit was then performed to determine the optimized intensity, position, and width of the peaks. The performance of the procedure was evaluated by means of the χ^2^ parameter [29]. Different normalizations to some peaks were performed in order to evidence the changes occurring at different doses and times of treatments with polyphenols. Useful information was also obtained by evaluating the ratio between the area of selected bands, as reported in Table 1.

Moreover, the band of Amide I (located in the 1550–1750 cm^−1^ wavenumber region) was analyzed in details since significant variations were expected due to protein configuration changes [30,31]. To determine protein secondary structure, the amide I band was deconvoluted by fitting it with mixed Lorentzian-Gaussian shaped components after localization of the minima in the second derivative spectrum These minima correspond to the positions of peaks within the band. In particular, second-derivative spectra were obtained using Savitsky-Golay derivative function algorithm for a seven data point window. The area of each absorption band was assumed to be proportional to the relative amount of the structure type in infrared spectra [32,33].

## 3. Results and Discussion

The univariate analysis previously described allowed us to use the $R^{2}$ parameter for evaluating the correlation between spectra from cell samples that underwent the same treatment (cells fixed at the same time after exposition and exposed to the same polyphenol dose). Its value ranges between 0.99 and 0.96, thus suggesting a high overall correlation. Conversely, the $R^{2}$ values obtained from the comparison of spectra from samples that underwent different treatments (different time of fixation, different polyphenol dose) are significantly lower, ranging from 0.75 to 0.82. After examining the $R^{2}$ values, we used the average spectra (evaluated by considering all the spectra obtained for samples in a given condition) for all of the analysis reported in the present paper.

### 3.1. Control Samples

**Figure 5:**
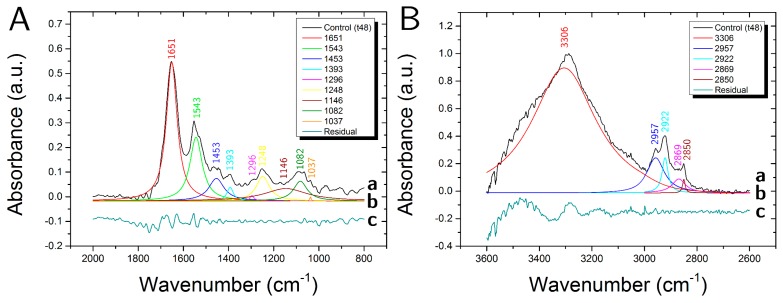
Average spectra of not exposed sample (**A**, (a)) in the range (2000–800 cm^−1^) and (**B**, (a)) (3600–2600 cm^−1^) with (b) deconvolution analysis of peaks with Lorentzian curves and (c) fit residual.

In Figure 5A,B the average spectra of a sample not exposed to the polyphenol extract are reported.

In the fingerprint region (1800–800 cm^−1^) (Figure 5A), different peaks that were representative of proteins and nucleic acids are clearly visible. In particular, the two strong bands centered at ≈1650 cm^−1^ and ≈1540 cm^−1^ are mainly attributed to the amide I (C=O and C-N) and amide II (N-H and C-N). The band at ≈1450 cm^−1^ is due to symmetric and asymmetric bending of the methylene groups (-CH_2_ and -CH_3_) and to -CH_2_ scissoring of proteins and lipids and the peak at ≈1400 is assigned to COO- groups asymmetric stretching of proteins and lipids. The two bands at ≈1240 cm^−1^ and ≈1085 cm^−1^ include, respectively, the asymmetric and symmetric -PO_2_ stretching vibrations of the phosphodiester nucleic acid backbone as well as contributions from C-O-P stretching of protein and lipids. The bands at ≈1150 and ≈1037 cm^−1^ are both assigned to a superposition of contribution arising from CO-O-C and C-O stretching vibrations of lipids, carbohydrates, and DNA. The high wavenumber region (HWR) from 3600 to 2800 cm^−1^ (Figure 5B) shows characteristic bands that are associated mainly with proteins, lipids and carbohydrates. In particular, the bands in the range 3100–3500 cm^−1^ are attributed to the amide A (-N-H) stretching motion of peptide backbones of proteins amino acids and O-H stretching of carbohydrate polysaccharides. The peaks observed at ≈2960 cm^−1^ and ≈2870 cm^−1^ are assigned, respectively, to the asymmetric and symmetric stretching of the methyl groups (-CH_3_) arising from cellular proteins and lipids. The bands at ≈2920 cm^−1^ and ≈2850 cm^−1^ are assigned, respectively, to the asymmetric and symmetric stretching of the methylene groups of membrane lipids (-CH_2_).

In Figure 5A,B, also the results of the deconvolution analysis of the two regions, (2000–800 cm^−1^) and (3600–2600 cm^−1^) are shown, they allow for us to identify less evident contributions from other molecular groups present in cell samples. In Table 2 the assignments for all the resolved peaks are reported.

### 3.2. Cells Exposed to PaDRw-25 Dose

The acquired spectra for samples that were exposed to the low dose of the investigated polyphenol extract left to act for 48 and 72 h (see Figures S1 and S2 in supplementary material) show similar features to the ones that were obtained for the control sample, as reported in Figure 5A,B, but they present some wavenumber shifts and some differences in peak intensity. 

In Table 3, the position of peaks for samples that were exposed to the low concentration of the polyphenol extract are reported for the two different exposure times. In some cases, the wavenumber shift (indicated with a bold character in Table 3) is higher than the spectral resolution available in our experiments. This is the case of Amide I (1651 cm^−1^), Amide II (1544 cm^−1^), Amide III (1296 cm^−1^), lipids (2869 and 1393 cm^−1^), DNA (1248 cm^−1^), and carbohydrates (1037 cm^−1^) contributions. The shifts are preserved for the two exposure times here investigated for Amide II, Amide III, lipids bands (2869 and 1399 cm^−1^), and DNA contributions (1248 and 1037 cm^−1^). Conversely, for Amide I, the wavenumber shift is below spectral resolution for 72 h exposure; for DNA band at 1082 cm^−1^, the wavenumber shifts is below spectral resolution for the 48 h exposure while it is higher for the 72 h exposure. These wavenumber shifts are indicative of changes in the lipid, protein and DNA characteristics of neuroblastoma cells.

**Figure 6:**
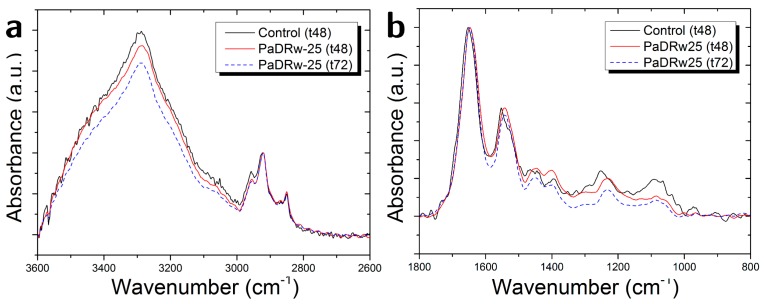
Comparison of average FT-IR spectra of the control cells and cells treated with a concentration of polyphenol extracts equal to 25 µg/mL fixed after 48 and 72 h of interaction in the region (**a**) (3600–2600 cm^−1^) and (**b**) (1800–800 cm^−1^).

Exposure to the low concentration of polyphenol extract also induces changes in the intensity of some peaks. This is illustrated in Figure 6a,b, where the average spectra for the control sample and for samples of cells that are exposed to PaDRw-25 for 48 and 72 h are shown. In Figure 6a, the spectra are normalized with respect to the 2922 cm^−1^ peak due to CH_2_ asymmetric stretching. In such a way, it is possible to note that polyphenol exposure causes a lowering of the Amide A and carbohydrates contribution increasing with exposure time and confirming a change in protein content similarly to the results reported in Ref. [14], where the decrease in Amide A band is related to the reduced contribution from glycogen OH absorption. In Figure 6b, the spectra are normalized with respect to Amide I band (1651 cm^−1^). This normalization enables us to note a decrease in the DNA region between 1300 and 1000 cm^−1^, and also an increase/decrease for 48/72 h exposure, respectively, around 1450 cm^−1^ that are generally attributed to lipid and protein contribution (see Table 2). The observed DNA decrease could be ascribed to the occurrence of apoptosis processes [4]. Our hypothesis is in line with data of Zelig et al. [37], who investigated FTIR’s utility for identifying and characterizing the different mode of cell death and established that DNA opaqueness to infrared light appears to be a uniquely useful parameter for discerning the death mode, since it decreases during apoptosis and increases during necrosis. Indeed, the degradation of nuclear DNA into nucleosomal units is broadly recognized as one of the hallmarks of apoptotic cell death [38]. 

**Figure 7:**
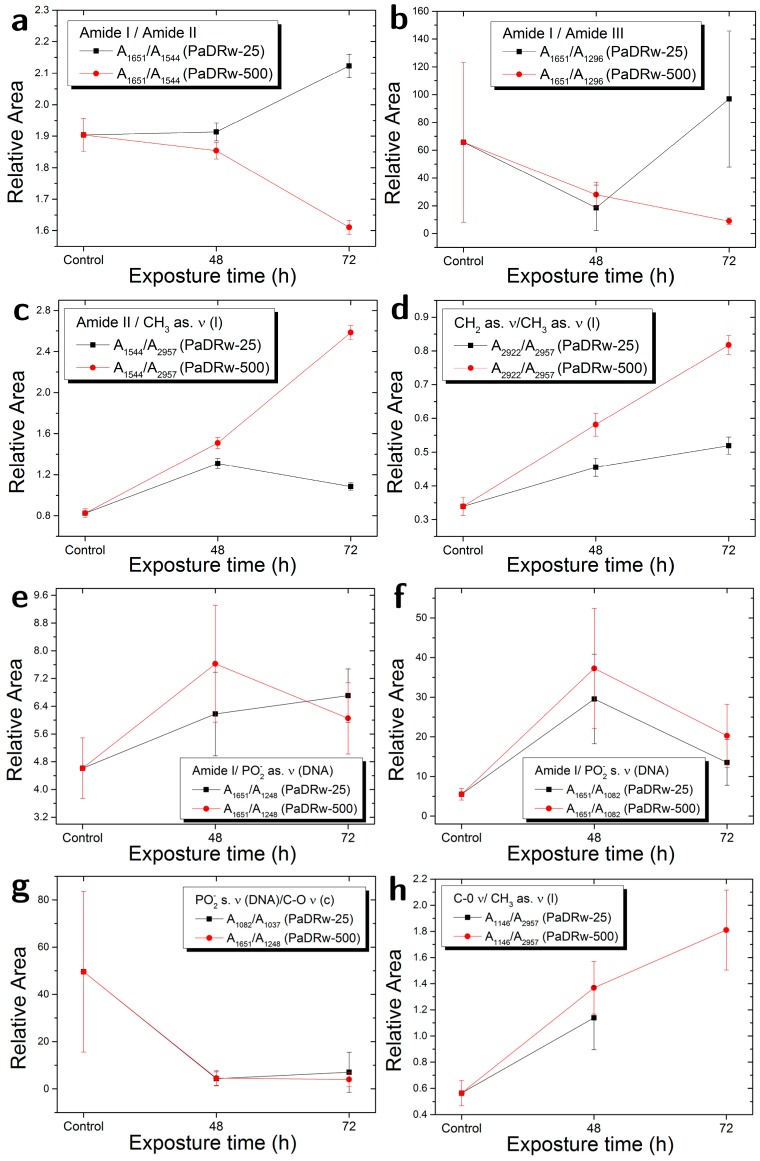

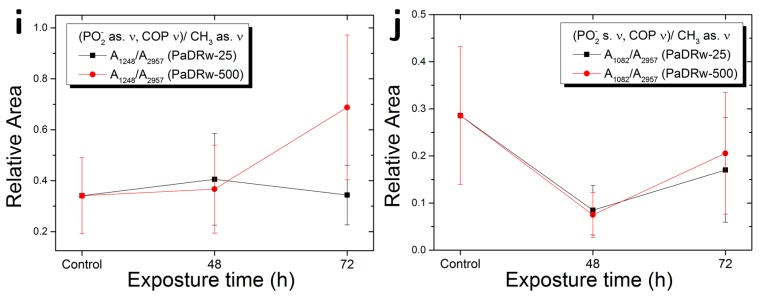
Comparison of the area ratios for selected peaks in FT-IR spectra collected at different times for samples exposed to a concentration of the PaDRw extract equal to 25 µg/mL (black) and to 500 µg/mL (red).

Additional information can be obtained when the behaviour of ratios between the area of some particularly significant bands in infrared spectra for the different exposure times are considered, as in Figure 7. In particular, in Figure 7a,b, the ratio between the area of Amide I and Amide II bands (A_1651_/A_1544_), and the one between Amide I and Amide III (A_1651_/A_1296_) band for the different exposure times show an increasing behaviour indicating changes in protein characteristics. The increase of A_1651_/A_1544_ can be attributed to a cleavage of cellular proteins, as reported in Ref. [4]. In Figure 7c, the ratio between the Amide II band and the band related to CH_3_ asymmetric stretching (A_1544_/A_2957_) due to lipid contribution is reported for the different exposure times. This ratio is generally used to evaluate the relative content of proteins and lipids. In Figure 7c, it is possible to note an increase in the 48 h exposure and a decrease in the 72 h exposure. This can suggest that there are stronger modifications in the initial phase of exposure and some attenuation effect in the successive times. In Figure 7d, the ratio between the areas of the bands that are related to CH_3_ and CH_2_ asymmetric stretching (A_2922_/A_2957_) is reported. In this case, the evidenced increasing trend, which indicated an increasing lipid saturation effect, strengthened further our hypothesis of apoptosis, whose occurrence is related to different membrane changes, such as phosphatidylserine exposure, membrane blebbing, and vesicle formation [37]. Indeed, it was found that apoptosis in neuronal cells has been associated with an increase in saturated fatty acid containing phospholipids [39]. These latter are the main structural components of membranes surroundings most intracellular organelles (e.g., lysosomes, endoplasmatic reticulum and nuclei), and are a major change in their composition could be expected to cause deformation and porosity of such membranes. As a result, an inflow of deoxyribonucleases into the nucleus occurs, causing the characteristic cleavage and laddering of chromosomal DNA observed during apoptosis. An increase by 10–20% in saturated fatty acids in a heterogeneous population of apoptotic and non-apoptotic cells was also suggested to be arise from a much sharper increase in saturated fatty acids only in the apoptotic cells [39]. Figure 7e,f report the ratio between the band due to Amide I and two different ones that are related to DNA contribution. In particular, in Figure 7e the A_1651_/A_1248_ values indicate an increase in the dependence of the interaction time. In Figure 7f, the A_1651_/A_1082_ values indicate a clear increase in 48 h exposure time samples and a value very near to the initial one for 72 h time samples. Figure 7e,f evidence changes in DNA content of exposed cells, in the first 48 h (see A_1651_/A_1248_ ratio ) and also in the 72 h time interval (see A_1651_/A_1082_ ratio), confirming the trend shown in Figure 6b. In Figure 7g, the A_1082_/A_1037_ ratio between DNA linked band and DNA/carbohydrates one is reported, the values show a similar decrease when compared to the control for both of the interaction times. In Figure 7h the A_1146_/A_2957_ ratio, which is linked to lipid peroxidation, shows an increase for the sample that is exposed to extract for 48 h (the peak at 1146 cm^−1^ for the 72 h exposure time is not resolved). Shibata et al. [40] screened lipid peroxidation products in SH-SY5Y cells, identifying 4-oxo-2-nonenal. This aldehyde, which is from the peroxidation of ω-6 polyunsaturated fatty acids, is an inducer of p53 phosphorylation and leads to the accumulation of protein and/or DNA damage, followed by apoptotic signalling that requires p53 in neuronal cells. Figure 7i,j report the A_1248_/A_2957_ and A_1082_/A_2957_ ratios, respectively, both of them are linked to protein phosphorylation [12]; the A_1248_/A_2957_ value in Figure 7i show no significant variations, while the A_1082_/A_2957_ value in Figure 7j show a slight decrease for the exposure time of 48 h.

### 3.3. Cells Exposed to PaDRw-500 Dose

The acquired spectra for the samples exposed to the high dose of PaDRw (see Figures S3 and S4 in supplementary material) show similar features to the one for the control sample reported in Figure 5A,B, but also they present some wavenumber shifts and some differences in peak intensity. 

In Table 4 the position of peaks for that were samples exposed to the high concentration of the polyphenol extract is shown for the two different exposure times. As for samples that are exposed to PaDRw-25, in some cases (indicated with a bold character in Table 4), the wavenumber shift is higher than the spectral resolution that is available for our measurements. This is the case of Amide I (1651 cm^−1^), Amide II (1544 cm^−1^), Amide III (1296 cm^−1^), lipids (2869 cm^−1^), DNA (1247 cm^−1^), and DNA/carbohydrates (1146 and 1037 cm^−1^) contributions. Differently from the case of samples that are exposed to the PaDRw low dose, these shifts are all preserved for the two exposure times here investigated and, as said before, are indicative of changes in the protein, lipid, and DNA characteristics of neuroblastoma cells.

**Figure 8:**
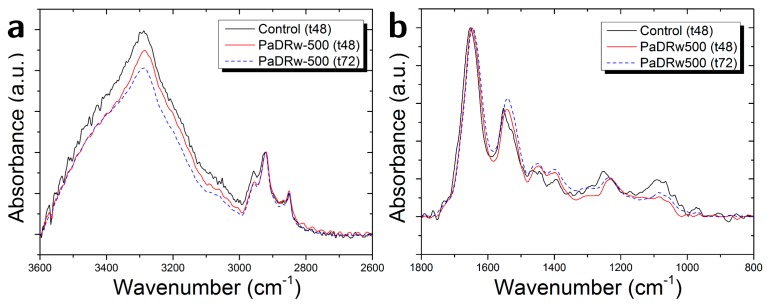
Comparison of average FT-IR spectra of the control cells and cells treated with a concentration of polyphenol extracts equal to 500 µg/mL fixed after 48 and 72 h of interaction in the region (**a**) (3600–2600 cm^−1^) and (**b**) (1800–800 cm^−1^).

The exposure to the high concentration of PaDRw also induces changes in the intensity of some peaks. This is evidenced in Figure 8a,b, where the average spectra for the control sample and for samples of cells that are exposed to PaDrw-500 for 48 and 72 h are shown. In Figure 8a, the spectra were normalized with respect to 2922 cm^−1^ peak due to CH_2_ asymmetric stretching. In such a way, it is possible to note that polyphenol exposure causes a lowering of Amide A and carbohydrates contribution, confirming a change in protein content, which increases with the exposure time, and a reduced contribution from glycogen OH absorption [14]. In the case of Figure 8b, the spectra were normalized with respect to Amide I band (1651 cm^−1^). This normalization allows for us to note a decrease in the DNA region between 1300 and 1000 cm^−1^, in agreement with previous literature data [4]. It is also evident of an increase around amide II and 1450 cm^−1^ region, whcih is generally attributed to lipid and protein contribution (see Table 2).

Also, in this case, the behaviour of the ratios between the area of some particularly significant bands in infrared spectra for the different exposure times (see Figure 7-red symbols) are considered in order to obtain more detailed information on the changes induced in SH-SyS5 cells by polyphenol extracts. A general view of 500 µg/mL values indicates that some of the considered ratios show different behaviours in comparison with the 25 µg/mL one, indicating that different doses of polyphenol extract can have different effects on neuroblastoma cells. In Figure 7a, the A_1651_/A_1544_ values for the different exposure times show a decreasing behaviour for 72 h samples, differently from the 25 µg/mL case, in which an increasing trend is observed. In Figure 7b, the A_1651_/A_1296_ ratio show a slight decrease for both the 48 and 72 exposure times. In these two graphs, the most important differences with the case of 25 µg/mL are present for the longest exposure time (72 h). In Figure 7c, the A_1544_/A_2957_ ratio between the Amide II band and the band related to CH_3_ asymmetric stretching due to lipid contribution shows an increasing behaviour for the different exposure times. In Figure 7d, the ratio between the area of the bands that are related to CH_3_ and CH_2_ asymmetric stretching (A_2922_/A_2957_) is reported, and, also, in this case, an increasing trend can be evidenced as for lower concentration. Figure 7e,f report the ratio between the band due to Amide I and two different ones related to DNA contribution. In particular, the A_1651_/A_1248_ values (Figure 7e) show an increase at 48 h exposure time and a decrease at 72 h, as well as the A_1651_/A_1082_ values (in Figure 7f). Also, for the high dose of the investigated polyphenol extract, Figure 7e,f point clear changes in DNA content of exposed cells, especially in the first 48 h, as observed for low polyphenol dose that can signify the presence of some apoptotic processes. In Figure 7g, the A_1082_/A_1037_ ratio between DNA linked band and DNA/carbohydrates one is reported, the A_1082_/A_1037_ values evidence a behaviour similar to that of the samples exposed to the PaDRw-25 with a decrease for both 48 and 72 h of exposure time. In Figure 7h, the A_1146_/A_2957_ ratio, which is linked to lipid peroxidation, shows an increase in the dependence of the exposure time, similarly to the trend that was observed for 25 µg/mL case. Figure 7i,j report the A_1248_/A_2957_ and A_1082_/A_2957_ ratios, respectively, both of them are linked to protein phosphorylation [12]. The A_1248_/A_2957_ value in Figure 7i shows an increase for the 72 h exposure time, while the A_1082_/A_2957_ value in Figure 7j shows a nearly constant behaviour with a slight decrease for the exposure time of 48 h. These trends indicate an increasing protein phosphorylation effect. Protein phosphorylation and dephosphorylation are known to be involved in the regulation of numerous cell functions. The treatment of SH-SY5Y with non-toxic doses of oxidant substances was found to alter the phosphorylation status of different phosphoproteins as an adaptive stress response that protects neuronal cells from oxidative stress [41].

### 3.4. Deconvolution of Amide I Band

**Figure 9:**
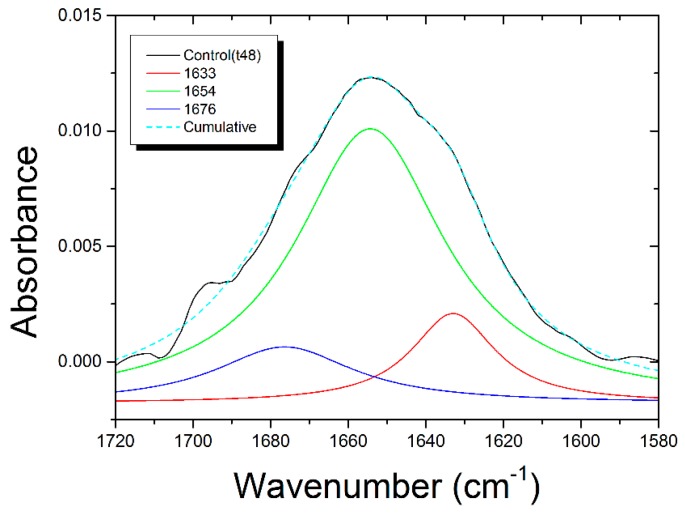
Deconvolution analysis of Amide I band for control cells.

As is well-known, the deconvolution analysis of the Amide I band observed in infrared spectra can give detailed information about the secondary structure of proteins and the conformational changes occurring as a result of interactions with external agents. In the present case, the Amide I band of control samples and of samples exposed to PaDrw-25 and PaDrw-500 at different time were analyzed using three subcomponents related to β-sheet (1633 cm^−1^), α-helix (1654 cm^−1^), and turn (1676 cm^−1^). In Figure 9, the results of the deconvolution procedure for the control spectrum is shown (see Figure S5 in supplementary material for the deconvolution results of extract treated samples).

The areas of different subcomponent bands are summarized in Table 5. It is possible to observe that the exposure to PaDRw induces a general increase in β-sheet contribution, a general decrease in α-helix subcomponent, and a limited decrease in turn component. In more detail, the increase of β-sheet contribution is stronger for a shorter exposure time and for PaDRw-500. A significant increase, also up to 75%, in β-sheet structures was previously associated with apoptosis [37]. Conversely, for α-helix subcomponent for PaDRw-25 dose, the decrease is similar for the two different exposure times, while for PaDRw-500, there is a marked decrease for 48 h exposure time and a partial recover for 72 h exposure time. The turn component decrease for PaDRw-25 at 48 h exposure time and has a partial increase for longer exposure time. For PaDRw-500 this component is fairly stable for 48 h of treatment and significantly decrease for longer exposure time.

This detailed analysis confirms that large changes occur in the secondary structure of Amide I band as a result of cells exposure to the investigated extract.

## 4. Conclusions

The results of the present investigation indicate that FT-IR spectroscopy is able to detect the changes that are occurring in SH-SY5Y neuroblastoma cells when they are exposed to an aqueous extract from sweet cherries that are rich in polyphenols. In particular, changes in protein, DNA, lipid, and carbohydrates are observed. The spectroscopic data show that different changes are induced by different doses of the extract and the effects are also dependent on the exposure time. The ratiometric approach shows interesting possibilities to highlight particular modification processes of the molecular structures in treated cells. The analysis of the deconvolution of Amide I band shows evidence of modification of the secondary protein structure following the action of the extract. The possibility to monitor in a detailed way the cell behaviour after polyphenols exposure using FT-IR spectroscopy is also of relevant interest in the perspective of introducing polyphenols derived substances in tumoral cells, in order to improve radiotherapy effectiveness. Further work is still in progress in order to make infrared spectroscopy an even more useful tool by using multivariate analysis and comparison with conventional cell assays (cell viability tests, assessment of mitochondrial redox activity inhibition and the Cytochalasin B-induced cytokinesis-block micronucleus assay). 

## Figures and Tables

**Table 1:** A_x_/A_y_ indicate the ratio between the areas of selected band [12,13,16,18]; abbreviation: as = asymmetric, s = symmetric, ν = stretching.

Ratio	Biomolecular Origin	Indication
A_X_ (cm^−1^)/A_Y_ (cm^−1^)		
A_1651_/A_1544_	Amide I/Amide II	Protein rearrangement,DNA/Protein content
A_1651_/A_1296_	Amide I/Amide III	Protein rearrangement
A_1544_/A_2957_	Amide II/CH_3_ as. ν	Protein/Lipid content
A_2922_/A_2957_	CH_2_ as. ν/CH_3_ as. ν	Lipid saturation
A_1651_/A_1248_	Amide I/PO_2_^−^ as. ν	Protein/DNA content
A_1651_/A_1082_	Amide I/PO_2_^−^ s. ν	Protein/DNA content
A_1082_/A_1037_	PO_2_^−^ s. ν/C-O ν	DNA/Carbohydrate content
A_1146_/A_2957_	C-O ν/CH_3_ as. ν	Lipid peroxidation
A_1248_/A_2957_	PO_2_^−^ as. Ν, C-O-P ν/CH_3_ as. ν	Protein phosphorylation
A_1082_/A_2957_	PO_2_^−^ s. ν, C-O-P ν/CH_3_ as. ν	Protein phosphorylation

**Table 2:** FT-IR peaks observed in the spectrum of control cells, with assignments in accordance with the data reported in the literature [12,13,14,26,30,34,35,36]; abbreviation: sym = symmetric, asym = asymmetric, str = stretching, ben = bending, sc = scissoring. The indicated position of every peak is the centre of the relative Lorentzian function obtained from the deconvolution fit.

Peak	Assignment			
cm^−1^	DNA/RNA	Protein	Lipid	Carbohydrate
3306		Amide A (N-H str.)		O-H str.
2957		CH_3_ asym. str.	CH_3_ asym. str.	
2922			CH_2_ asym. str.	
2869		CH_3_ sym. str.	CH_3_ sym. str.	
2850			CH_2_ sym. str.	
1651		Amide I (C=O str., C-N str.)		
1544		Amide II (C-N str., C-NH ben.)		
1453		CH_3_ asym. ben., CH_2_ sc.	CH_3_ asym. ben., CH_2_ sc.	
1393		COO^−^ sym. str.	COO^−^ sym. str.	
1296		Amide III		
1248	PO_2_^−^ asym. str.	C-O-P str.	C-O-P str.	
1146	CO-O-C str.		C-O str.	C-O str., CO-O-C str.
1082	PO_2_^−^ sym. str.	C-O-P str.	C-O-P str.	
1037			C-O str.	C-O str.

**Table 3:** FT-IR peaks position for control and samples treated with 25 µg/mL of crude extract (PaDRw) extract and fixed after 48 and 72 h of interaction. The shift in terms of units of wavenumber are indicated in brackets (bold values stand for shifts greater than the spectral resolution of the instrument 4 cm^−1^). Abbreviations: p = proteins, l = lipids, c = carbohydrates.

Control	PaDRw-25 (t48)	PaDRw-25 (t72)
Peak (cm^−1^)	Peak (cm^−1^)	Peak (cm^−1^)
3306 (p, c)	3308 (+2)	3308 (+2)
2957 (p, l)	2958 (+1)	2958 (+1)
2922 (l)	2921 (−1)	2922
2869 (p, l)	2874 (**+5**)	2875 (**+6**)
2850 (l)	2850	2851 (+1)
1651 (p)	1646 (**−5**)	1648 (−3)
1544 (p)	1538 (**−6**)	1538 (**−6**)
1453 (p, l)	1450 (−3)	1449 (−4)
1393 (p, l)	1399 (**+6**)	1398 (**+5**)
1296 (p)	1305 (**+9**)	1304 (**+8**)
1248 (DNA)	1231 (**+17**)	1230 (**+18**)
1146 (DNA, c)	1150 (+4)	-
1082 (DNA)	1084 (+2)	1096 (**+14**)
1037 (DNA, c)	1057 (**+20**)	1067 (**+30**)

**Table 4:** FT-IR peaks position for control and samples treated with 500 µg/mL of PaDRw extract and fixed after 48 and 72 h of interaction. The shift in terms of units of wavenumber are indicated in brackets (bold values stand for shifts greater than the spectral resolution of the instrument 4 cm^−1^). Abbreviations: p = proteins, l = lipids, c = carbohydrates.

Control	PaDRw-500 (t48)	PaDRw-500 (t72)
Peak (cm^−1^)	Peak (cm^−1^)	Peak (cm^−1^)
3306 (p, c)	3301 (**−5**)	3313 (**+7**)
2957 (p, l)	2958 (+1)	2959 (+2)
2922 (l)	2921 (−1)	2922
2869 (p, l)	2878 (**+9**)	2874 (**+5**)
2850 (l)	2851 (+1)	2851 (+1)
1651 (p)	1646 (**−5**)	1644 (**−7**)
1544 (p)	1537 (**−7**)	1536 (**−8**)
1453 (p, l)	1448 (**−5**)	1450 (−3)
1393 (p, l)	1397 (+4)	1396 (+3)
1296 (p)	1303 (**+7**)	1307 (**+11**)
1248 (DNA)	1231 (**+17**)	1233 (**+15**)
1146 (DNA, c)	1157 (**+11**)	1129 (**−17**)
1082 (DNA)	1084 (+2)	1085 (+3)
1037 (DNA, c)	1056 (**+19**)	1056 (**+19**)

**Table 5:** Amide I deconvolution results for control and samples treated with PaDrw-25 and PaDRw-500.

Control	Assignments	PaDRw-25 (t48)	PaDRw-25 (t72)	PaDRw-500 (t48)	PaDRw-500 (t72)
Peak (cm^−1^)		Peak (cm^−1^)	Peak (cm^−1^)	Peak (cm^−1^)	Peak (cm^−1^)
1633	Β-sheets	1634 (+1)	1634 (+1)	1634 (+1)	1630 (−3)
%A = 10 ± 3		%A = 34 ± 2	%A = 31 ± 5	%A = 38 ± 4	%A = 26 ± 3
1654	Α-helix	1654	1654	1654	1652 (−2)
%A = 52 ± 13		%A = 30 ± 3	%A = 31 ± 7	%A = 22 ± 5	%A = 42 ± 5
1676	Turn	1678 (+2)	1675 (−1)	1677 (+1)	1678 (+2)
%A = 10 ± 4		%A = 6.1 ± 0.9	%A = 8 ± 2	%A = 11 ± 2	%A = 3.8 ± 1.0

## Supplementary Materials

The following are available online at www.mdpi.com/2079-6374/7/4/57/s1.
