# Supplementary material for: Study of SH-SY5Y Cancer Cell Response to Treatment with Polyphenol Extracts Using FT-IR Spectroscopy
Source: Biosensors (Basel). 2017 Nov 30;7(4):57. doi: 10.3390/bios7040057 (PMC5746780; doi:10.3390/bios7040057)
Supplement: Supplementary file 1 [file biosensors-07-00057-s001.pdf]

biosensors

Supplementary Materials: Study of SH-SY5Y cancer cells response to treatment with polyphenol extracts using FT-IR spectroscopy

Valerio Ricciardi, Marianna Portaccio, Simona Piccolella, Lorenzo Manti, Severina Pacifico and Maria Lepore

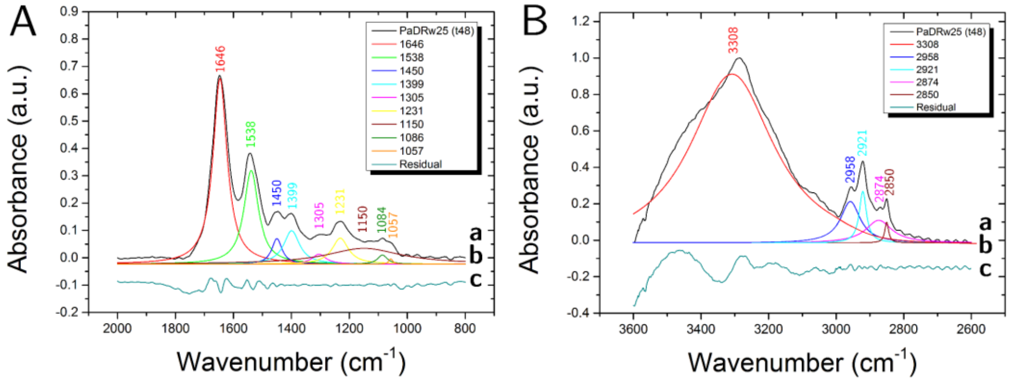

Figure S1. Average spectra of sample treated with a dose of 25 µg/ml of PaDRw for 48 hours (**A**, **a**) in the range [2000–800 cm^{-1}] and (**B**, **a**) [3600–2600 cm^{-1}] with (**b**) deconvolution analysis of peaks with Lorentzian curves (**c**) and fit residual.

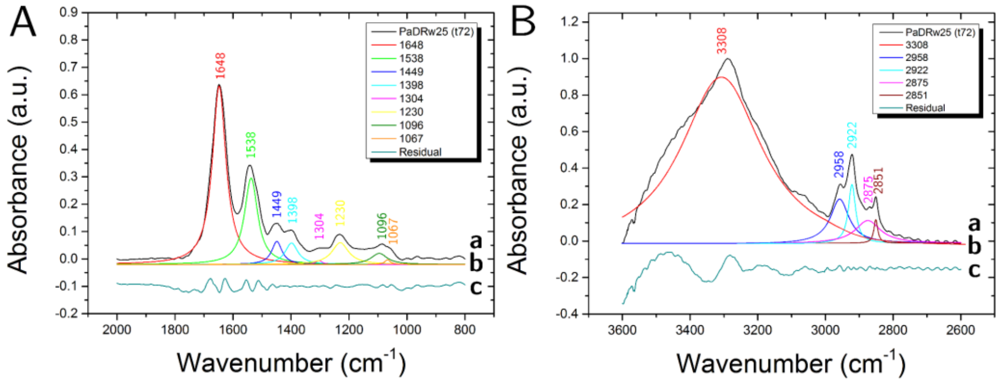

Figure S2. Average spectra of sample treated with a dose of 25 µg/ml of PaDRw for 72 hours (**A**, **a**) in the range [2000–800 cm^{-1}] and (**B**, **a**) [3600–2600 cm^{-1}] with (**b**) deconvolution analysis of peaks with Lorentzian curves and (**c**) fit residual.

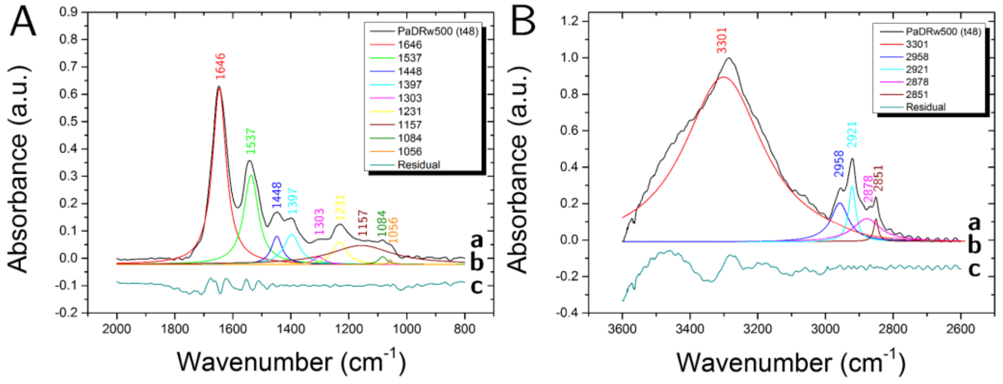

Figure S3. Average spectra of sample treated with a dose of 500 μg/ml of PaDRw for 48 hours (A, a) in the range [2000–800 cm^{-1}] and (B, a) [3600–2600 cm^{-1}] with (b) deconvolution analysis of peaks with Lorentzian curves and (c) fit residual.

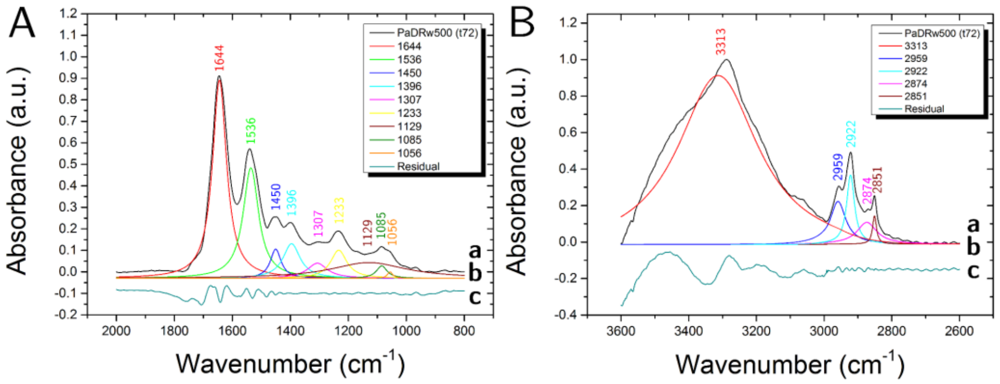

Figure S4. Average spectra of sample treated with a dose of 500 μg/ml of PaDRw for 72 hours (**A**, **a**) in the range [2000–800 cm^{-1}] and (**B**, **a**) [3600–2600 cm^{-1}] with (**b**) deconvolution analysis of peaks with Lorentzian curves and (**c**) fit residual.

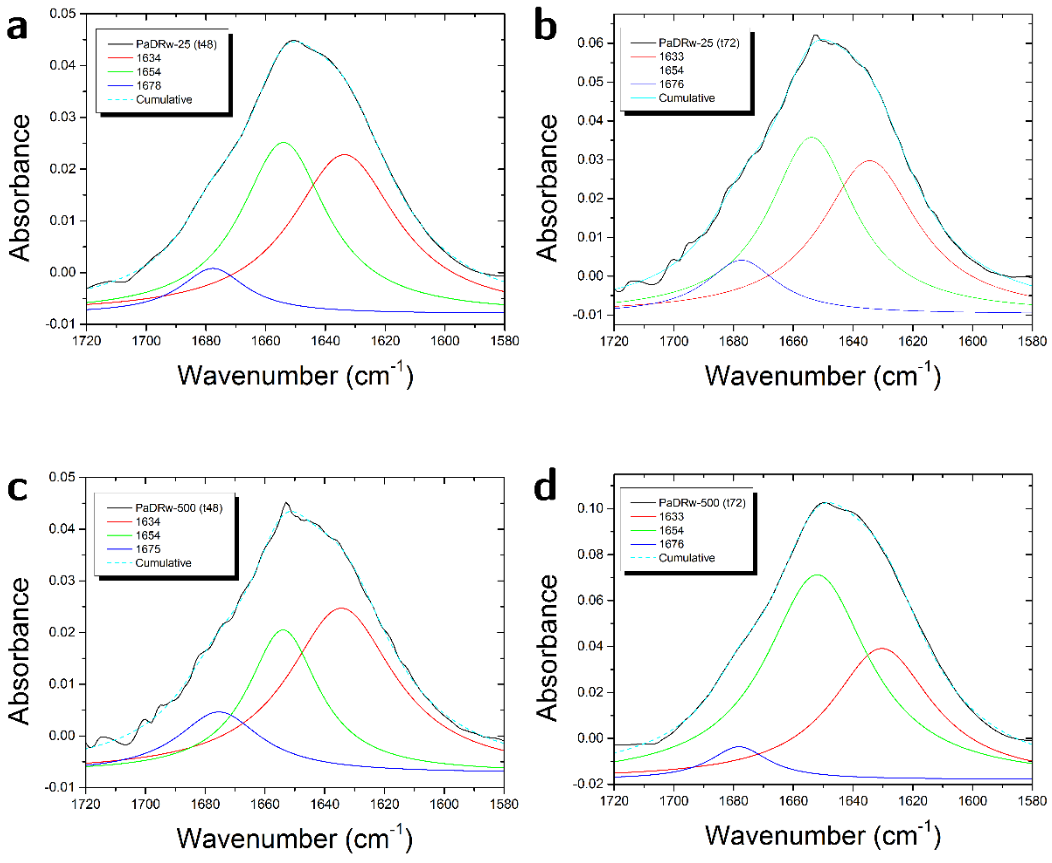

Figure S5. Deconvolution analysis of Amide I band (**a**) for cells exposed to the low dose of polyphenol extract at 48 h and (**b**) 72 h; (**c**) and (**d**) the same for cells exposed to the high dose of polyphenol extract.

© 2017 by the authors. Submitted for possible open access publication under the terms and conditions of the Creative Commons Attribution (CC BY) license (http://creativecommons.org/licenses/by/4.0/).
